# The CC-V Flap: A Novel Technique for Augmenting a C-V Nipple Reconstruction Using a Free Dermal Graft

**Published:** 2014-01

**Authors:** Sarah Elizabeth Clark, EPL Turton

**Affiliations:** The Breast Unit, St. James University Hospital, Leeds, UK

**Keywords:** Nipple reconstruction, CV flap, Dermal graft, Patient satisfaction

## Abstract

**BACKGROUND:**

We present a novel method for augmenting the standard C-V flap used for nipple reconstruction with a free dermal graft which aims to improve the appearance of the nipple reconstruction, decrease loss of projection and improve patient satisfaction overall.

**METHODS:**

The surgical technique for performing a free dermal graft augmentation of a CV flap is described. All patients who underwent this technique between February 2009 and January 2012 at our unit were contacted by telephone, questioned about any complications and asked to rate their satisfaction with the technique.

**RESULTS:**

In a series of 18 nipple reconstructions, there were no immediate post-operative complications and long term follow up shows that that this technique achieves high patient satisfaction scores.

**CONCLUSIONS:**

The CC-V flap is a safe technique which scores highly with patients for cosmetic appearance after long term follow up.

## INTRODUCTION

There are many methods available for reconstructing the nipple and one of the most commonly employed is the C-V flap. However, a common complaint post-operatively is gradual loss of projection of the reconstruction over time. Several methods have been described to overcome this. Eo *et al.*^1^ describe augmenting the C-V flap with a dermofat graft taken whilst performing simultaneous contra-lateral breast reduction for symmetrisation. In a series of 20 cases, there were no post-up complications and although they state projection was maintained, no long term follow up data has been published. Jamnadas-Khoda and colleagues^2^ have described the “cigar roll” flap as a modification of the CV flap. In this technique one of the V flaps is de-epithelialised and rolled under the 2^nd^ V flap. However the complication rate in their series of 50 patients was high at 10%. Macdonald *et al.*^3^ presented a similar modification to the C-V flap which they called the “Swiss-roll” flap but only reported a series of 3 cases making assessment of outcomes impossible. Other methods of augmenting the C-V flap using conchal cartilage^4^ and silicon rods^5^ have been reported but also had high rates of complications. We present a method to augment the C-V flap with a free dermal graft which we have called a composite C-V flap (CC-V flap) which to our knowledge has not been previously described in the literature.

## MATERIALS AND METHODS

All patients who underwent a CC-V flap reconstruction between February 2009 and January 2012 were identified from the Breast Unit database. All patients underwent nipple reconstruction at least 6 months after Latissimus Dorsi (LD) or implant based breast reconstruction. A telephone interview was undertaken in which patients were asked if they thought their nipple reconstruction had lost projection over time. If so, they were asked to quantify this as 25%, 50% or 75% loss of projection. They were asked to score the projection out of 10 with 10 being sufficient projection and 0 being no projection at all. They were also asked to score the nipple reconstruction out of 10 for the overall cosmetic appearance. The patients’ opinion of the nipple reconstruction and if they considered it a worthwhile procedure was also recorded. Ethical approval was not required for this study but all patients verbally consented to participate and gave written consent for photographs to be used.

**Fig. 1:**
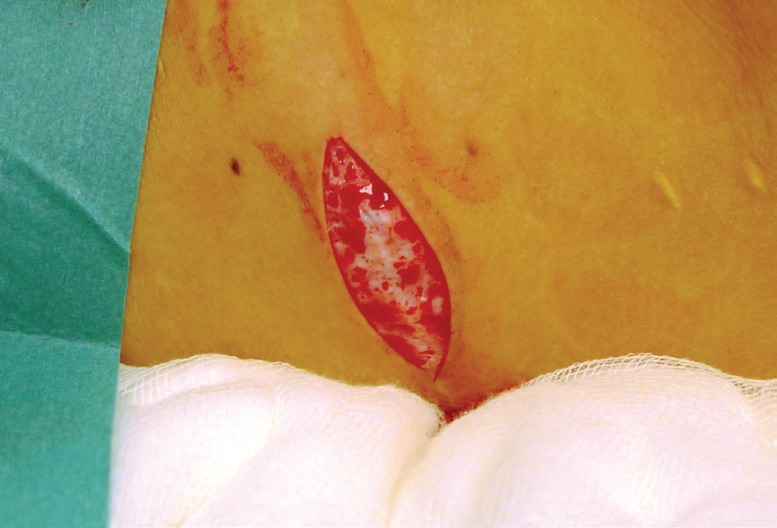
Donor site

The most suitable donor sites for the dermal graft were lateral dog ears from LD or TRAM/DIEP reconstruction scars but if these were not available skin can be taken from the axilla. An elliptical incision was made and a very fine de-epithelialisation performed. We used a 23 blade for this. This was shown in Figure 1. The remaining dermal ellipse was then excised with any subcutaneous fat required to achieve a flat closure of the donor site. The graft was kept in saline until it was ready to be prepared. Haemostasis was performed and the donor site was closed with a 3/0 monocryl interrupted deep dermal suture and a 4/0 monocryl sub-cuticular suture.

**Fig. 2:**
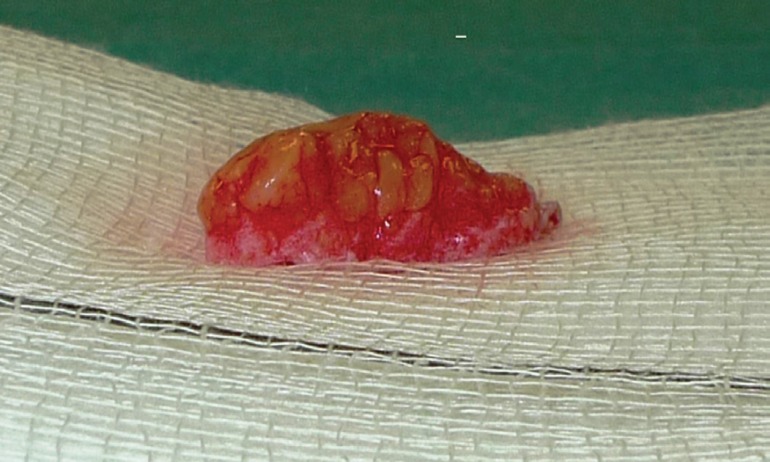
Graft removed

**Fig. 3:**
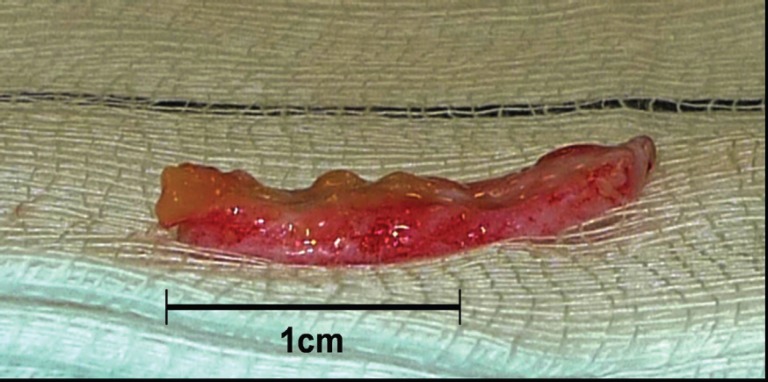
Subcutaneous fat removed from graft

**Fig. 4:**
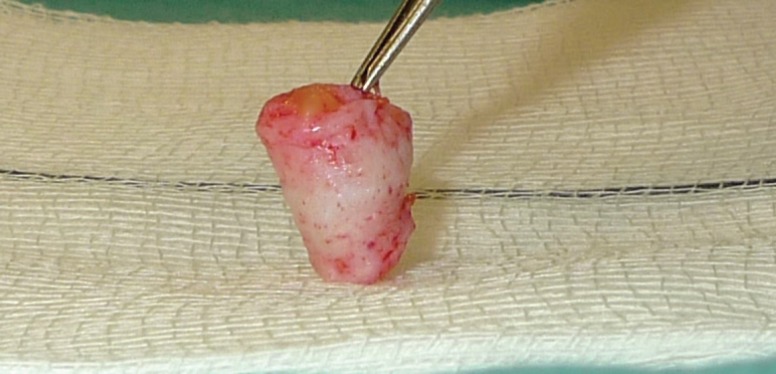
Graft rolled up and secured in a roll.

The graft was prepared by removing any subcutaneous fat with Strabismus scissors leaving a flap purely made of dermis. The graft size was adjusted as required. This could be seen in Figure 2 and 3. The flap was the rolled up like a cigar with the de-epithelialized side kept externally as illustrated in Figure 4. It was secured with two 4/0 vicryl rapide sutures. It was then returned to the saline until ready for use.

**Fig. 5:**
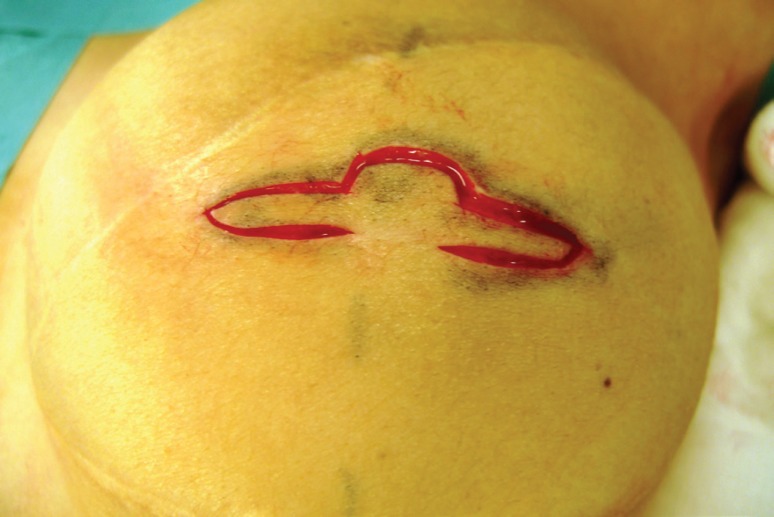
C-V flap cut out of reconstructed breast skin

**Fig. 6:**
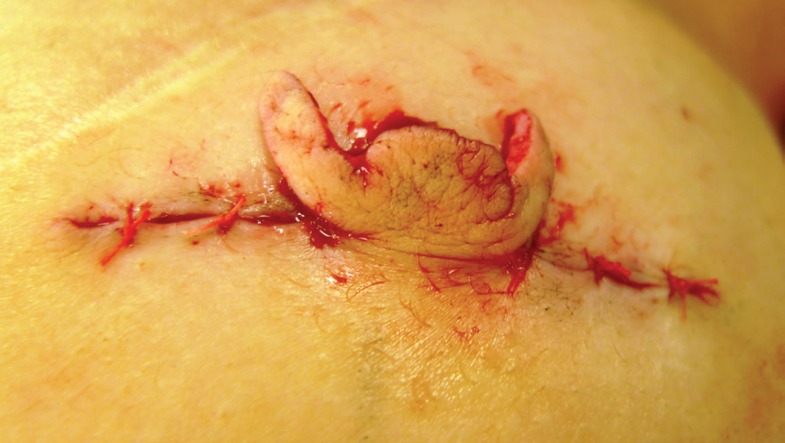
Wings of C-V flap brought round to form a tube

**Fig. 7:**
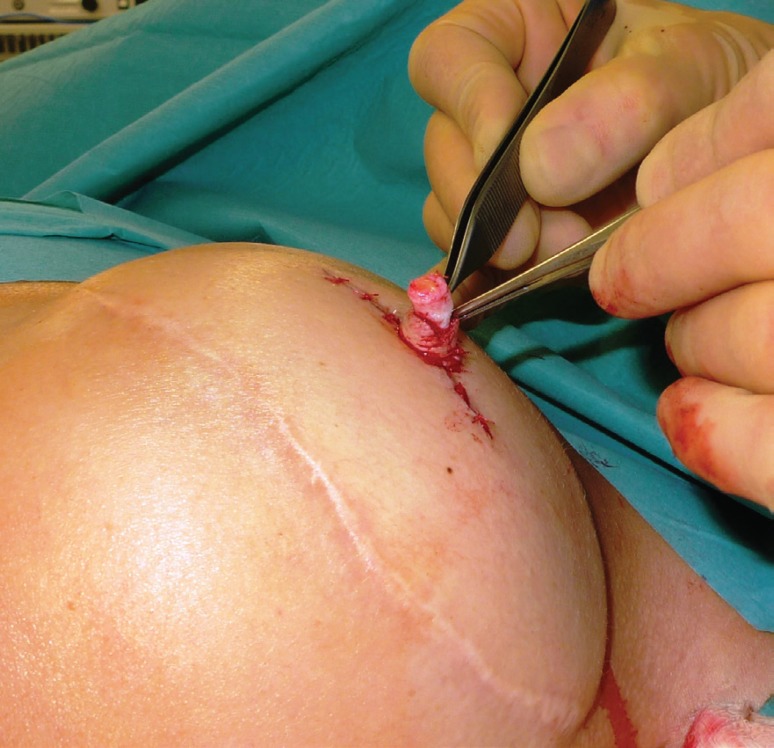
Graft inserted into tube of C-V flap

**Fig. 8:**
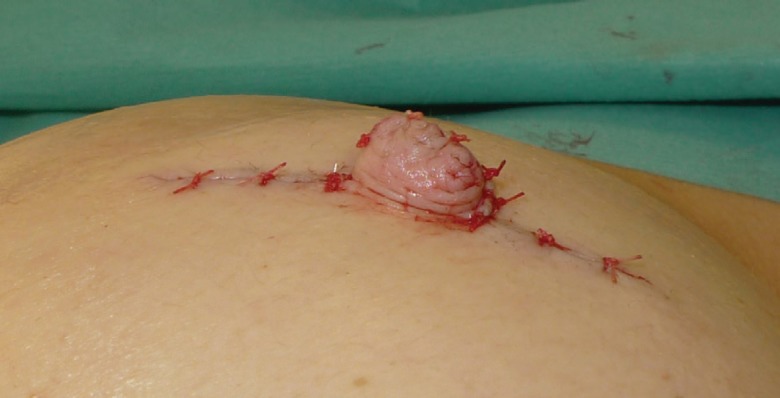
Completed CC-V flap

The C-V nipple reconstruction was performed using the traditional method. A full thickness dermal flap was raised as shown in Figure 5. Haemostasis was performed with bipolar forceps. The linear incision was closed with 3/0PDS deep dermal sutures and the skin closed with interrupted 4/0 vicryl rapide. The lateral wings of the C-V flap were wrapped round to form the base and sutured in the standard way with 4/0 vicryl rapide as seen in Figure 6. The free dermal graft was then inserted into the tube formed by the two wings which was illustrated by Figure 7. The lid of the nipple reconstruction was then closed over the top of the graft and sutured into position. The final result was shown in Figure 8.

**Fig. 9:**
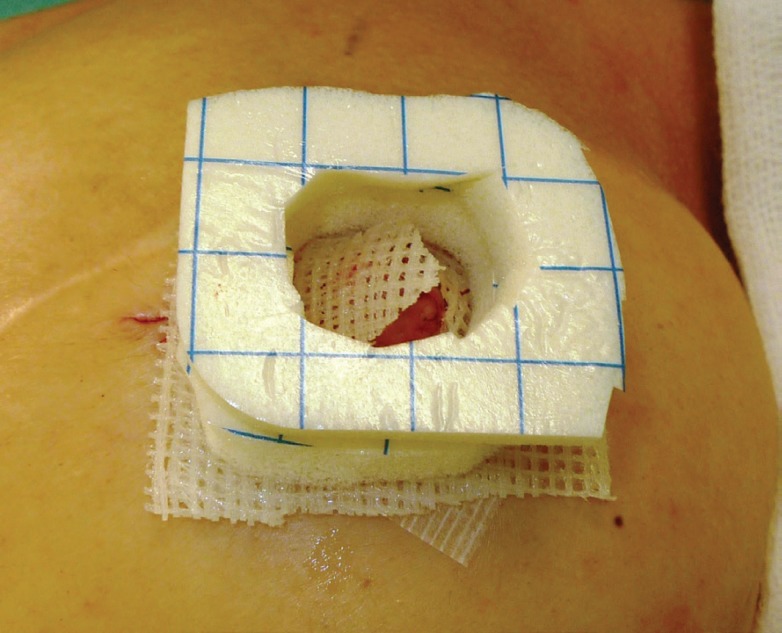
Lyofoam dressing

**Fig. 10:**
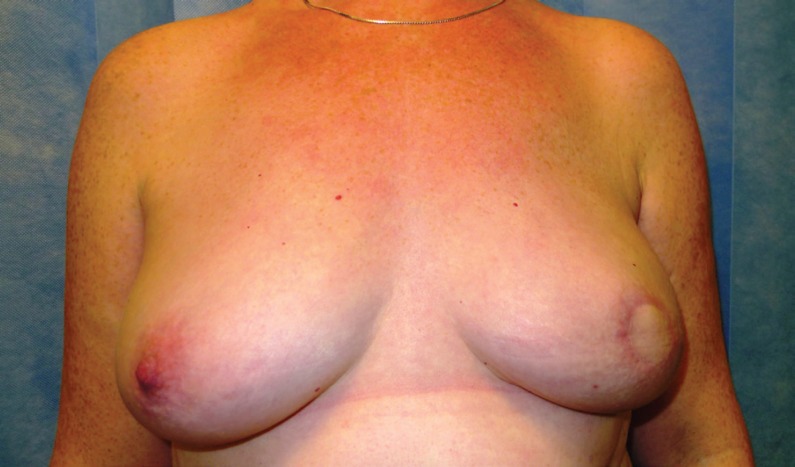
Patient with Left autologous LD reconstruction prior to CC-V flap nipple reconstruction

**Fig. 11:**
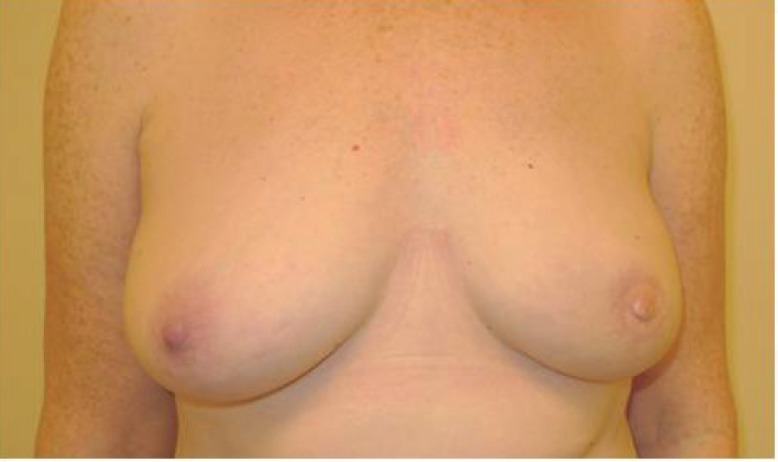
Patient with Left autologous LD reconstruction after CC-V flap nipple reconstruction

A protective dressing was important and kept in place for 2 weeks. Steristrips were applied to the linear wound. A loose piece of Jelonet with a split was laid over the nipple reconstruction. Two pieces of Lyofoam had a central hole cut into them and were placed gently over the nipple reconstruction to protect it as seen in Figure 9. A waterproof dressing was then applied loosely over the top. Patient with left autologous LD reconstruction prior to CC-V flap nipple reconstruction was shown in Figure 10. Figure 11 demaonstrats the patient with left autologous LD reconstruction after CC-V flap nipple reconstruction. 

The steristrips and Jelanet were removed at the first post-operative visit 2 weeks after surgery and the patients were then advised to continue using the Lyofoam circles in their bra to continue protecting the reconstruction for the next 4 weeks.

## RESULTS

20 patients underwent CC-V flap nipple reconstruction between February 2009 and January 2012. Of these 4 had bilateral procedures and 3 were re-do operations after a previous C-V flap had loss of all projection. Fourteen were able to be contacted by telephone, 1 patient had died and 5 patients were unreachable. The results were based on the 14 patients who undertook the telephone interview and correspond to 18 CC-V flap nipple reconstructions.

The average time since nipple reconstruction was 36.8 months (range=19-54 months). The majority of patients had undergone an autologous LD reconstruction (10/23), one had LD+ implant reconstruction, six had implant based reconstruction and one had an oncoplastic reconstruction of the breast following a central excision. There was one smoker in the cohort and five patients had undergone radiotherapy prior to nipple reconstruction. There were no immediate post-operative complications. Three patients reported no loss of projection. Of those that reported loss of projection one estimated it to be 25%, eight as 50%, four as 75% and 2 as 100% loss. The average score out of 10 for nipple projection was 4.6 (range=1-10). There was no association between previous radiotherapy and loss of projection. The average score for overall cosmetic appearance was 7.3 out of 10 (range=4-10). Thirteen of the fourteen patients felt the procedure had been worthwhile and had improved the overall appearance of their breast reconstruction.

## DISCUSSION

We are aware that this is a small number of cases but in our series the complete lack of immediate post-operative complications shows this to be a safe technique which heals well. A long term evaluation of outcomes of 252 C-V flap nipple reconstructions by Otterburn *et al.*^6^ reported an overall complication rate of 4% (3.2% were tip necrosis and 0.8% wound dehiscence). Some degrees of loss of projection were reported by 83% of patients in our series with the majority estimating that about half of the nipple projection had been lost over time. The average score out of 10 for overall appearance was 7.3 in this series. These results compared favourably with reports in the literature of patient satisfaction rates between 67 and 81%.^6^^-^^8^ Loksen *et al.*^7^ reported 42% of patients were satisfied with nipple projection at 5.53 years follow-up in 14 C-V flap reconstructions. Valdetta *et al.*^8^ reported an average patient satisfaction score of 6.28 out of 10 for projection at 1 year in 29 C-V flap reconstructions. In Otterburn *et al.*’s series of 252 C-V flaps the average patient satisfaction score for nipple projection was 3.2 out of 5, though 38% of patients desired more projection. The limitation of our study is that nipple projection immediately post-operatively and at last follow-up appointment was not measured and the degree of flattening has been estimated by the patients. However, it is how the patients feel about their nipple reconstruction, rather than measurements which were ultimately important. Patients who underwent nipple reconstruction after breast reconstruction tend to have higher rates of satisfaction compared to those who did not^9^^.^ All but one of our patients who had undergone nipple reconstruction was glad they had it and felt it to be a worthwhile technique and would have it done again. Interestingly, the one patient who did not feel it had been worthwhile gave the highest scores for projection and overall appearance. Most of the studies mentioned above have also found that patients are satisfied with appearance even if the nipple has lost projection. The CC-V flap is a safe technique which scores highly with patients for cosmetic appearance after long term follow up.

## Footenote

Please cite this paper as:

Clark SE, Turton E. The CC-V Flap: A Novel Technique for Augmenting a C-V Nipple Reconstruction Using a Free Dermal Graft. World J Plast Surg 2014;3(1):8-12.
